# Outcome Assessment in Children and Adolescents With Chronic Pain: An International Clinical Practice Survey

**DOI:** 10.1002/ejp.70216

**Published:** 2026-01-17

**Authors:** Jordi Miró, Elisabet Sánchez‐Rodríguez, Mark P. Jensen, Christina Liossi, Susan M. Lord, Inese Gobina, Nathan Skidmore, Mary O'Keeffe, Susan M. Walker, Rocío de la Vega, Pablo Ingelmo, Helen Koechlin, Minna Ståhl, Jennifer Stinson, Rikard K. Wicksell, G. Allen Finley, Jesús Cebrecos, Liesbet Goubert, Francisco Reinoso‐Barbero, Daniela C. Rosenberger, Esther M. Pogatzki‐Zahn

**Affiliations:** ^1^ Pediatric Pain Division Universitat Rovira i Virgili Tarragona Spain; ^2^ Centre de Recerca en Avaluació i Mesura de la Conducta Universitat Rovira i Virgili Tarragona Spain; ^3^ Institut Investigacions Sanitàries Pere Virgili Universitat Rovira i Virgili Tarragona Spain; ^4^ Department of Rehabilitation Medicine University of Washington Seattle Washington USA; ^5^ Department of Psychology University of Southampton Southampton UK; ^6^ School of Medicine and Public Health The University of Newcastle Callaghan Australia; ^7^ Department of Education and Research Children's Clinical University Hospital Latvia Riga Latvia; ^8^ Institute of Public Health Riga Stradins University Riga Latvia; ^9^ Department of Sport and Exercise Sciences Durham University Durham UK; ^10^ UCD School of Public Health, Physiotherapy and Sports Science University College Dublin Belfield, Dublin 4 Ireland; ^11^ Developmental Neurosciences UCL Great Ormond Street Institute of Child Health London UK; ^12^ Department of Psychology Universidad de Málaga Málaga Spain; ^13^ Instituto de Investigación Biomédica de Málaga y Plataforma en Nanomedicina (IBIMA Plataforma BIONAND) Malaga Spain; ^14^ Department of Medicine and Surgery Università degli Studi di Milano‐Bicocca Milan Italy; ^15^ Department of Psychosomatics and Psychiatry, University Children's Hospital University of Zurich Zurich Switzerland; ^16^ Division of Child and Adolescent Health Psychology, Department of Psychology University of Zurich Zurich Switzerland; ^17^ Children's Research Centre, University Children's Hospital Zurich University of Zurich Zurich Switzerland; ^18^ Finnish Centre for Children and Adolescent Pain Management and Research HUS New Children's Hospital Helsinki Finland; ^19^ The Hospital for Sick Children Research Institute Toronto Canada; ^20^ Department of Clinical Neuroscience Karolinska Institutet Stockholm Sweden; ^21^ Pain Clini Capio S:t Göran Hospital Stockholm Sweden; ^22^ IWK Health Centre Halifax Canada; ^23^ ESTEVE Healthcare Barcelona Spain; ^24^ Department of Experimental‐Clinical and Health Psychology Ghent University Ghent Belgium; ^25^ Pediatric Pain Unit Hospital La Paz Madrid Spain; ^26^ Department of Anaesthesiology, Intensive Care and Pain Medicine University Hospital Muenster Muenster Germany

**Keywords:** chronic pain, outcome assessment (health care)/standards, paediatrics, pain measurement, patient reported outcome measures, surveys and questionnaires

## Abstract

**Background:**

Effective treatment of paediatric chronic pain requires a robust and comprehensive set of outcome assessment tools to evaluate treatment effectiveness. Although a core outcome set (COS) exists for clinical trials, its practicability and appropriateness for clinical practice is currently unknown. This cross‐sectional study led by the IN‐ChildPain group aimed to: (1) identify clinical outcome domains and measures used by clinicians treating children and adolescents with chronic pain, (2) determine which domains are considered mandatory in clinical routine and (3) compare prioritisation across disciplines and countries.

**Methods:**

An online survey, available in eight languages, was conducted eliciting data from clinicians who treat children and adolescents with chronic pain. Percentages of the most commonly used outcomes were calculated, and *z*‐tests were performed to compare study variables based on participants' country income status and professional background.

**Results:**

A total of 193 clinicians from 42 countries participated. The most commonly assessed domains were pain intensity (84%), pain interference (80%) and physical functioning (79%), with higher assessment rates in high‐income countries. Pain intensity and interference were deemed mandatory by 93% of participants, followed by physical functioning (92%). However, only 53% reported using patient‐reported outcome measures, with the 0–10 Numeric Rating Scale being the most common (94%). Assessment practices varied as a function of country income level and professional background.

**Conclusions:**

The findings highlight the need to develop and implement a COS tailored to the needs and resources of clinicians. Such standardisation would enhance consistency in assessment, enable cross‐site benchmarking and promote equitable pain care globally.

**Significance:**

This study provides critical insights into how clinicians assess paediatric chronic pain, highlighting significant global disparities and professional differences in outcome domain prioritisation. By identifying commonly assessed domains, these findings emphasise the need for standardised measures and pave the way for developing a core outcome set tailored to clinical activities. Such an advance is essential for improving the consistency and quality of care for children and adolescents with chronic pain worldwide.

## 1 Introduction

The prevalence of chronic pain in children and adolescents is high (Chambers et al. 2024) and has gradually increased during the last three decades (Roy et al. 2022; Ståhl et al. 2014). About 5% of children experience high‐impact chronic pain (Huguet and Miró 2008), which profoundly disrupts their physical, emotional and social functioning (Miró et al. 2023; Roman‐Juan et al. 2024; Solé et al. 2024). Despite its impact, chronic pain in children and adolescents remains largely underrecognised and undertreated (Eccleston et al. 2021). An incomplete understanding of the mechanisms and impact of chronic pain, and a lack of standardised use of patient‐reported outcome measures across different clinical settings, limit the development and evaluation of treatment options.

In 2005, the paediatric working group of the Initiative on Methods, Measurement and Pain Assessment in Clinical Trials (PedIMMPACT) sought to improve chronic pain assessment by identifying key domains to measure, and recommending specific measures for use in clinical trials (McGrath et al. 2008). Despite these efforts, clinical and research practice remained largely unchanged (Connolly et al. 2019). In 2021, an expert group developed a new set of key outcome domains to address limitations identified in the PedIMMPACT recommendations. This initiative represented a significant advancement, as it incorporated input from patients, parents and clinicians to define a new set of outcome domains for clinical trials through a Delphi process (Palermo et al. 2021), and a new set of respective measures (Palermo et al. 2024). Although these initiatives sought input from clinicians and individuals with lived experience to enhance clinical relevance, they were primarily designed for structured clinical trials. The extent to which their recommendations can be applied in everyday clinical practice remains largely unknown. Moreover, clinical trials often take place under idealised conditions that do not reflect the realities of clinical practice, where time and resources may be limited. Further, clinical trials frequently apply strict inclusion criteria, excluding patients with comorbidities and other complexities, which further limits their applicability to real‐world settings (Singal et al. 2014). This discrepancy was shown in a study comparing adult participants in randomised controlled trials (RCTs) for chronic primary musculoskeletal pain with patients treated at a large university hospital. The exclusion criteria used in RCTs closely mirrored the comorbidities observed in clinical practice, particularly psychiatric conditions (Koechlin et al. 2025). Hence the movement towards pragmatic clinical trials that bridge the gap between traditional research settings and the complexities of real‐world pain care is highly needed (Roman‐Juan et al. 2023). To facilitate pragmatic trials, it is essential to determine the outcome domains and identify their measures that are relevant in clinical practice and feasible within clinical workflows across a range of real‐world settings and countries (Salvat et al. 2021). What may be feasible in the controlled environment of an RCT may not be practical in everyday practice and workflows.

Given these considerations and to advance towards a COS that enables best clinical practice with children and adolescents with chronic pain, this study aimed to improve our understanding of the domains and measures currently used in paediatric chronic pain clinical practice. Specifically, we aimed to (1) identify the outcome domains and measures currently used, (2) determine which domains clinicians perceive as mandatory, and (3) compare domain prioritisation across professions and countries. In doing so, this study directly addresses a critical gap: the extent to which outcome domains and measures defined for paediatric chronic pain clinical trials can be applied and operationalised in real‐world clinical settings.

## 2 Methods

### 2.1 The International Network on Chronic Pain in Childhood

In August 2022, the ERA‐NET NEURON (https://www.neuron‐eranet.eu/) announced a call for proposals to establish networks focused on chronic pain research. In response, a proposal to create an international, interdisciplinary network dedicated to chronic pain in childhood (The International Network on Chronic Pain in Childhood; IN‐ChildPain) was submitted. This proposal was approved in April 2023. The IN‐ChildPain network aims to identify critical knowledge gaps, develop recommendations to address these gaps and provide guidelines to support future research about chronic pain in children and adolescents. IN‐ChildPain comprises 24 experts, primarily from Europe, with additional representation from Australia, Canada and the United States (https://www.neuron‐eranet.eu/projects/INCHILDPAIN/). The network brings together clinicians and researchers across multiple disciplines, alongside representatives from patient advocacy groups, the pain‐related industry and the European Pain Federation (EFIC; https://europeanpainfederation.eu/).

IN‐ChildPain identified the potential mismatch between core outcome sets (COS) designed for research and their utility in clinical practice as a key issue. The consortium developed a survey study to explore this potential mismatch in detail. By identifying gaps and overlaps, the survey sought to provide insights into whether a separate COS should be developed to meet the distinct needs of clinical practice as opposed to the needs of traditional research, thereby facilitating a broader effective implementation and utilisation in paediatric pain management, benchmarking and pragmatic trials. The survey is reported following the Checklist for Reporting Results of Internet E‐Surveys (CHERRIES; Eysenbach 2004).

### 2.2 Survey Design and Setting

A cross‐sectional survey was conducted online using LimeSurvey, a web‐based survey platform, from May 2024 to November 2024. It was available in eight languages (i.e., Arabic, Catalan, English, French, Italian, Portuguese‐Brazil, Portuguese‐Portugal and Spanish). The survey items were developed based on input from the authorship team. The survey was piloted with a group of 10 clinicians and researchers in paediatric chronic pain to identify potential difficulties, with no changes implemented prior to the broader survey launch. Translation was performed by project partners and EFIC chapter members. Each translated version included the same list of outcome domains and concise definitions adapted from the PedIMMPACT (McGrath et al. 2008) and Palermo et al. (2021) frameworks to ensure conceptual consistency across languages. An English copy of the survey is provided as Appendix S1.

### 2.3 Participant Inclusion Criteria and Recruitment

The survey was distributed to potential participants (i.e., healthcare professionals who work in the treatment of chronic pain in children and adolescents) identified by various procedures: (1) individuals listed as the contact person of a programme for managing chronic pain in children and adolescents in the ‘Global list of pediatric chronic pain programs’ managed by Dr. Tonya Palermo available from IASP Pain in Childhood SIG (http://childpain.org/wp‐content/uploads/2025/01/PedPainClinicList_2025_12.23.2024.pdf; although the exact number of paediatric pain programmes worldwide is unknown, this registry represents the most comprehensive source currently available); (2) individuals identified by members of the IN‐ChildPain network as a key person in a programme not included in the previous list; in addition (3) the survey was also disseminated widely across Africa, Europe, Latin America, North‐America and Southeast Asia with the help of the following organisations: the Paediatric Pain South Africa Special Interest group (PAESPAINSA), the European Pain Federation (EFIC), the Latin American Federation of Associations for the Study of Pain (*Federación Lationamericana de Asociaciones para el Estudio del Dolor*; FEDELAT), the Association of Southeast Asian Pain Societies (ASEAPS), the Lebanese Society for the Study of Pain and the PAEDIATRIC‐PAIN email listserv hosted at Dalhousie University in Halifax, Canada. No incentive was provided for participation. Only healthcare professionals could proceed in the survey, as respondents were required to confirm their clinical role and to report their professional background in the first section. This ensured that all included participants were clinicians involved in paediatric pain care.

To ensure the integrity of the survey data, duplicate entries from the same individual were systematically prevented by restricting users with the same IP address from accessing the survey multiple times. Additionally, a manual review was conducted to identify and remove any potential duplicates from the dataset. This process involved cross‐referencing the programme names and demographic information provided by participants. Two authors (E.S.‐R. and J.M.) independently reviewed all entries for duplicates, with a third reviewer available for adjudication if needed; however, no such intervention was required. The review confirmed that there were no duplicate entries in the database.

### 2.4 Ethics and Consent

The Ethical Committee concerning Research into People, Society and the Environment (CEIPSA) of the *Universitat Rovira i Virgili* approved the study procedures (ref.: CEIPSA‐2024‐PR‐0020). Potential participants were required to provide informed consent. Once they consented to participate, they were allowed to enter the survey website, read the questions and respond. Informed consent was obtained through an introductory information page describing the study's aims, voluntary participation, confidentiality and data protection. Proceeding to the survey implied consent, consistent with accepted ethical standards for online surveys.

### 2.5 Survey Procedure/Administration

The survey was openly available and took < 5 min to complete. No responses were excluded based on the time taken to complete the survey. Once participants consented to participate in the study, they were asked if they were healthcare professionals involved in treating children or adolescents with chronic pain. Those who responded ‘No’ were exited from the survey, while those who responded ‘Yes’ proceeded to a 10‐question survey divided into four sections, each presented on a separate screen. The questions were presented in a fixed order, and participants were allowed to review or modify their responses using a ‘back’ button. The first section contained four questions to elicit basic information about the chronic pain programme and the respondents, including the country where the pain programme was located, the type of services offered, the name of the pain clinic or programme, and the respondent's professional background. The second section focused on how the pain outcome assessments were conducted in in‐ and outpatient programmes for the management of chronic pain in children and adolescents and included four questions about the domains assessed and the questionnaires used to assess those domains. Specifically, respondents were asked to indicate: (1) the outcome domains their programmes assessed using patient self‐reported questionnaires, (2) which domains they thought should be ‘mandatory’ or ‘optional’ in clinical practice, and (3) the Patient‐Reported Outcome Measures (PROMs) used in their programmes, selected from a list that included the domains and PROMs identified by the PedIMMPACT (McGrath et al. 2008) and Palermo and colleagues (Palermo et al. 2021, 2024) groups, with additional domains that our expert group deemed relevant. Moreover, respondents were also asked to report (4) any other standardised questionnaires they used that were not included in the list provided. The third section included a single question asking whether the respondent's pain programme was participating in a chronic pain registry, with those responding affirmatively prompted to specify the registry name. The final section invited participants to indicate their potential interest in future research collaborations and offered the option to provide their email address for potential follow‐up.

### 2.6 Data Analysis

We used descriptive statistics (counts and percentages) to summarise demographic and outcome data, and *z*‐tests to compare percentages of the study variables considering the participants' country income status (i.e., high‐, upper‐middle‐ and lower‐middle‐income countries, according to the World Bank classifications; https://datahelpdesk.worldbank.org/knowledgebase/articles/906519‐world‐bank‐country‐and‐lending‐groups) and professional background. The number of respondents in most professional background categories was too low for reliable comparisons. Therefore, two decisions were made: (1) to group the responses from participants who identified as general practitioners (GP) and paediatricians, based on the rationale that paediatricians could be considered as GPs for younger populations, and (2) to limit comparisons to professional categories with more than 5% of the responses. Consequently, comparisons were conducted between the categories including anaesthesiologists, general practitioners and paediatricians, psychologists, and pain physicians. No adjustments (e.g., item weighting or propensity scores) were applied. All analyses were performed using the SPSS statistics package, version 29.0 (SPSS Inc., Chicago, Illinois, USA).

## 3 Results

A total of 440 individuals accessed the introductory page of the survey and reviewed the participant information sheet. Of these, 324 provided consent to participate, resulting in a participation rate of 74%. However, 90 participants did not meet the inclusion criteria of being a healthcare professional involved in the treatment of children or adolescents with chronic pain, and 20 did not respond to this eligibility question. As a result, 214 participants proceeded to the survey questions. Of these, 193 completed at least two of the three survey sections (90%), and 185 completed the entire survey (86%). Table 1 provides an overview of the participant clinicians' country of practice organised as a function of World Bank country income level. Table 2 presents participants' professional backgrounds, and Table 3 outlines the services offered in their clinics or centres, and how many centres participate in clinical registries.

**TABLE 1:** Participants by country and World Bank country income level (*N* = 193).

	*N* (%)
High‐income country	83 (43%)
Australia	2 (1%)
Belgium	1 (< 1%)
Canada	6 (3%)
Chile	8 (4%)
Germany	3 (1%)
Finland	4 (2%)
France	1 (< 1%)
Hungary	1 (< 1%)
Ireland	1 (< 1%)
Italy	1 (< 1%)
Malta	1 (< 1%)
Netherlands	2 (1%)
New Zealand	1 (< 1%)
Panama	2 (1%)
Portugal	6 (3%)
Romania	1 (< 1%)
Saudi Arabia	5 (3%)
Singapore	1 (< 1%)
Slovenia	1 (< 1%)
Spain	10 (5%)
Sweden	1 (< 1%)
Switzerland	1 (< 1%)
United Kingdom	8 (4%)
United States	14 (7%)
Uruguay	1 (< 1%)
Upper‐middle‐income country	61 (32%)
Argentina	2 (1%)
Brazil	3 (1%)
Colombia	5 (2%)
Cuba	19 (10%)
Dominican Republic	2 (1%)
Ecuador	5 (2%)
Indonesia	1 (< 1%)
Mexico	8 (4%)
Moldova	1 (< 1%)
Peru	11 (5%)
South Africa	3 (1%)
Turkey	1 (< 1%)
Lower‐middle‐income country	43 (22%)
Bolivia	39 (20%)
Egypt	1 (< 1%)
Lebanon	2 (1%)
Nigeria	1 (< 1%)
No data about income level	6 (3%)
Venezuela^a^	6 (3%)

^a^ Venezuela is considered by the World Bank as unclassifiable.

**TABLE 2:** Participants by professional background (*N* = 164^a^).

	*N* (%)
Anaesthesiologist	82 (50%)
General practitioner	16 (10%)
Psychologist	12 (7%)
Pain physician	9 (5%)
Paediatrician	9 (5%)
Physical medicine and rehabilitation	8 (5%)
Rheumatologist	7 (4%)
Physiotherapist	3 (2%)
Nurse	3 (2%)
Neurologist	3 (2%)
Traumatologist	3 (2%)
Oncologist	3 (2%)
Surgeon	2 (1%)
Natural and traditional medicine	2 (1%)
Occupational therapist	1 (< 1%)
Palliative medicine	1 (< 1%)

^a^ Twenty‐nine participants skipped this question.

**TABLE 3:** Type of services offered by the participants' clinics or centres and participation in clinical registries (*N* = 193).

	*N* ^a^
Inpatient intensive pain rehabilitation programme	45
Outpatient service associated with a hospital	144
Outpatient service in a private practice	57
Clinics or centres participating in a clinical registry	19

^a^ Some clinics/centres had two or more services, so the total *N* will be higher than 193.

Responses were collected from participants located in 42 countries and 193 centres across five continents. According to World Bank income classifications, 83 centres (43%) were in high‐income countries, 61 (32%) in upper‐middle‐income countries and 43 (22%) in lower‐middle‐income countries. Notably, six centres (3%) were from Venezuela, considered by the World Bank as unclassifiable. Therefore, data from participants from Venezuela were included in calculations for global indexes but excluded from country‐level comparisons. Most participants were physicians, representing a variety of specialties, including anaesthesiologists, general practitioners, pain specialists and paediatricians, among others. The category ‘pain specialist’ refers to clinicians with formal training or certification in pain medicine, regardless of their primary medical specialty. Overall, the study sample included individuals from 16 distinct professional profiles, as detailed in Table 2. As can be seen in Table 3, most of the centres (*N* = 144; 75%) provided pain management as an outpatient service.

### 3.1 Outcome Domains Assessed in Clinical Practice

The outcome domains that participants reported assessing in their programmes for chronic pain are presented in Table 4. The most frequently assessed domains were pain intensity (*N* = 163, 84%), pain interference (*N* = 154, 80%) and physical functioning (*N* = 152, 79%), although some variations were observed across country income categories. For example, in the upper‐middle‐income category, emotional functioning was assessed more commonly than physical functioning (*N* = 44, 72%; *N* = 43, 71%, respectively), while in the lower‐middle‐income category, pain severity was assessed more commonly than physical functioning (*N* = 29, 67%; *N* = 26, 60%, respectively).

**TABLE 4:** Outcome domains assessed in the chronic pain programmes as a function of participants' country income (*N* = 193).

	Total, *N* (%)	High income, *n* (%)	Upper‐middle‐income, *n* (%)	Lower‐middle‐income, *n* (%)
	193 (100%)	83 (100%)	61 (32%)	43 (22%)
Pain intensity	163 (84%)	75 (90%)^a^	53 (87%)^b^	30 (70%)^a,b^
Pain interference	154 (80%)	77 (93%)^c,d^	46 (75%)^c^	28 (65%)^d^
Physical functioning	152 (79%)	79 (95%)^e,f^	43 (71%)^e^	26 (60%)^f^
Pain severity	147 (76%)	72 (87%)^g,h^	43 (71%)^g^	29 (67%)^h^
Emotional functioning	139 (72%)	68 (82%)^i^	44 (72%)^j^	23 (53%)^i,j^
Sleep quality	138 (72%)	70 (84%)^k,l^	41 (67%)^k,m^	22 (51%)^l,m^
Overall well‐being	127 (66%)	58 (70%)	42 (69%)	24 (56%)
Global judgement of treatment satisfaction	108 (56%)	44 (53%)	40 (66%)	22 (51%)
Fatigue	100 (52%)	50 (60%)^n^	32 (53%)	16 (37%)^n^
Adverse events	98 (51%)	45 (54%)^o^	36 (59%)^p^	15 (35%)^o,p^
Cognitive functioning	92 (48%)	41 (49%)	31 (51%)	16 (37%)
Role functioning	89 (46%)	50 (60%)^q,r^	24 (39%)^q^	14 (33%)^r^
Economic factors	60 (31%)	23 (28%)^s^	27 (44%)^s,t^	8 (19%)^t^

*Note:* High income: *N* = 83 (43%); upper‐middle‐income: *N* = 61 (32%); lower‐middle‐income: *N* = 43 (22%).

^a^ *z* = 2.94; *p* = 0.002; ^b^ *z* = 2.14; *p* = 0.016; ^c^ *z* = 2.92; *p* = 0.002; ^d^ *z* = 3.95; *p* < 0.001; ^e^ *z* = 4.07; *p* < 0.001; ^f^ *z* = 4.96; *p* < 0.001; ^g^ *z* = 2.40; *p* = 0.008; ^h^ *z* = 2.58; *p* = 0.005; ^i^ *z* = 3.38; *p* < 0.001; ^j^ *z* = 1.96; *p* = 0.025; ^k^ *z* = 2.42; *p* = 0.008; ^l^ *z* = 3.98; *p* < 0.001; ^m^ *z* = 1.65; *p* = 0.050; ^n^ *z* = 2.45; *p* = 0.007; ^o^ *z* = 2.06; *p* = 0.020; ^p^ *z* = 2.42; *p* = 0.008; ^q^ *z* = 2.48; *p* = 0.007; ^r^ *z* = 2.95; *p* = 0.002; ^s^ *z* = −2.06; *p* = 0.020; ^t^ *z* = 2.73; *p* = 0.003.

Fewer than half of the participants reported assessing cognitive functioning (*N* = 92, 48%), role functioning (*N* = 89, 46%) and economic factors (*N* = 60, 31%). Statistically significant differences were observed when comparing responses across country income categories. High‐income countries reported significantly higher rates of assessment across nearly all domains, compared to upper‐middle‐ and lower‐middle‐income countries (see Table 4). At the same time, upper‐middle‐income countries generally reported higher assessment rates than lower‐middle‐income countries. For example, in high‐income countries, at least six of the 13 listed outcome domains were assessed in 75% of the cases. In comparison, only two of the 13 domains met this threshold in upper‐middle‐income countries and none in lower‐middle‐income countries.

### 3.2 Outcome Domains Perceived as Mandatory by Clinicians

Table 5 presents the outcome domains perceived as mandatory in clinical settings, in total and separately for the different country income levels. Overall, pain intensity (*N* = 180, 93%) and pain interference (*N* = 180, 93%) emerged as the most frequently identified domains deemed essential by participants, closely followed by physical functioning (*N* = 177, 92%). Among the various income categories, pain intensity was considered mandatory by nearly all upper‐middle‐ and lower‐middle‐income countries (*N* = 59, 97% and *N* = 43, 100%, respectively), whereas high‐income countries placed slightly less emphasis on pain intensity (*N* = 72, 87%). Conversely, role functioning and economic factors were regarded as mandatory by a smaller segment of participants (*N* = 97, 50% and *N* = 86, 45%, respectively), indicating less consensus about their importance.

**TABLE 5:** Outcome domains perceived as mandatory as a function of participants' country income level (*N* = 193).

	Total, *N* (%)	High income, *n* (%)	Upper middle‐income, *n* (%)	Lower middle‐income, *n* (%)
Pain intensity	180 (93%)	72 (87%)^c,d^	59 (97%)^c^	43 (100%)^d^
Pain interference^a^	180 (93%)	81 (98%)^e^	58 (95%)^f^	35 (81%)^e,f^
Physical functioning^b^	177 (92%)	78 (94%)	55 (90%)	39 (91%)
Pain severity^a^	170 (88%)	69 (83%)^g^	53 (87%)^h^	42 (98%)^h,h^
Emotional functioning^b^	170 (88%)	76 (92%)^i^	50 (82%)^i^	39 (91%)
Overall well‐being^a^	165 (85%)	69 (83%)	54 (88%)	38 (88%)
Sleep^b^	160 (83%)	69 (83%)	51 (84%)	36 (84%)
Global judgement of treatment satisfaction	157 (81%)	61 (73%)^j,k^	54 (88%)^j^	38 (88%)^k^
Adverse events^a^	129 (67%)	46 (55%)^l^	52 (85%)^l,m^	26 (60%)^m^
Cognitive functioning	129 (67%)	50 (60%)^n^	45 (74%)^n^	31 (72%)
Fatigue	126 (65%)	51 (61%)	40 (66%)	31 (72%)
Role functioning	97 (50%)	49 (59%)^o^	29 (47%)	18 (42%)^o^
Economic factors	86 (45%)	24 (29%)^p,q^	36 (59%)^p^	23 (54%)^q^

*Note:* High income: *N* = 83 (43%); upper‐middle‐income: *N* = 61 (32%); lower‐middle‐income: *N* = 43 (22%).

^a^ Proposed as mandatory and ^b^optional domains by Palermo et al. (2021).

^c^z = −2.06*; ^d^ *z* = −2.50**; ^e^ *z* = 3.19***; ^f^ *z* = 2.24*; ^g^ *z* = −2.39**; ^h^ *z* = −1.93*; ^i^ *z* = 1.72*; ^j^ *z* = −2.22*; ^k^ *z* = −1.93*; ^l^ *z* = −3.79***; ^m^ *z* = 2.87**; ^n^ *z* = −1.69*; ^o^ *z* = 1.83*; ^p^ *z* = −3.62***; ^q^ *z* = −2.70**.

*p < 0.05; **p < 0.01; ***p < 0.001.

Statistically significant differences were found when comparing the rates of outcomes perceived as mandatory across countries in different income categories (see Table 5). Moreover, the outcome domains perceived as mandatory differed significantly based on the respondents' professional background (see Table 6). Specifically, most outcome domains identified as mandatory by psychologists differed significantly from those reported by anaesthesiologists and/or general practitioners and paediatricians. However, psychologists and pain physicians differed only in their perception of the outcome domain ‘adverse events’, which was deemed mandatory by 78% of pain physicians but by 0% of the psychologists (*z* = −3.74, *p* < 0.001). In contrast, no significant differences were observed between the groups of general practitioners and paediatricians and pain physicians.

**TABLE 6:** Outcome domains perceived as mandatory as a function of participants' professional background (*N* = 193).

	Anaesthesiology, *N* (%)	General practitioner and paediatrician, *N* (%)	Psychology, *N* (%)	Pain physician, *N* (%)
	82 (100%)	25 (100%)	12 (100%)	9 (100%)
Pain intensity	79 (96%)^a^	24 (96%)	10 (83%)^a^	8 (89%)
Pain interference	79 (96%)^b^	21 (84%)^b^	12 (100%)	8 (89%)
Physical functioning	74 (90%)	22 (88%)	12 (100%)	9 (100%)
Pain severity	74 (90%)^c^	22 (88%)^d^	7 (58%)^c,d^	7 (78%)
Emotional functioning	70 (85%)	21 (84%)	12 (100%)	9 (100%)
Sleep	73 (89%)^e,f^	18 (72%)^e^	7 (58%)^f^	8 (89%)
Overall well‐being	71 (87%)^g^	20 (80%)	7 (58%)^g^	8 (89%)
Global judgement of treatment satisfaction	72 (88%)^h,i^	21 (84%)^j^	5 (42%)^h,j^	6 (67%)^i^
Fatigue	55 (67%)	15 (60%)	6 (50%)	5 (56%)
Adverse events	70 (85%)^k,l^	13 (52%)^k,m^	0 (0%)^l,m,n^	7 (78%)^n^
Cognitive functioning	57 (69%)^o^	15 (60%)^p^	3 (25%)^o,p^	5 (56%)
Role functioning	39 (48%)	12 (48%)	5 (42%)	4 (44%)
Economic factors	43 (52%)	14 (56%)^q^	1 (8%)^q^	3 (33%)

^a^ *z* = 1.87, *p* = 0.030; ^b^ *z* = 2.18, *p* = 0.014; ^c^ *z* = 2.99, *p* = 0.001; ^d^ *z* = 2.05. *p* = 0.020; ^e^ *z* = 2.09, *p* = 0.018; ^f^ *z* = 2.79, *p* = 0.003; ^g^ *z* = 2.43, *p* = 0.008; ^h^ *z* = 3.88, *p* < 0.001; ^i^ *z* = 1.72, *p* = 0.043; ^j^ *z* = 2.64, *p* = 0.004; ^k^ *z* = 3.50. *p* < 0.001; ^l^ *z* = 6.33, *p* < 0.001; ^m^ *z* = 3.10, *p* < 0.001; ^n^ *z* = −3.74, *p* < 0.001; ^o^ *z* = 3.00, *p* = 0.001; ^p^ *z* = 1.99, *p* = 0.023; ^q^ *z* = 2.77; *p* = 0.003.

*p < 0.05; **p < 0.01; ***p < 0.001.

### 3.3 Patient‐Reported Outcome Measures (PROMs) Used in Clinical Practice

Of the 193 participants, 103 (53%) reported the use of PROMs in their clinical practice. Among these, 54 participants were from high‐income countries (HIC), representing 65% of respondents from HIC. In addition, 31 participants using PROMs were from upper‐middle‐income countries (UMIC), being 51% of respondents from UMIC, and 15 participants were from lower‐middle‐income countries (LMIC) or 35% of respondents from LMIC. Additionally, three participants from Venezuela (i.e., 50% of Venezuelan respondents) also reported routinely using PROMs.

Statistically significant differences were found in the use of PROMs between HIC and both UMIC (*z* = 1.72, *p* = 0.043) and LMIC (*z* = 3.23, *p* < 0.001). As shown in Table 7, among the various PROMs presented in the survey, the 0–10 Numeric Rating Scale was the only measure widely used, with 94% of participants reporting its use. In contrast, all other PROMs were used by fewer than half of the sample. A complete list of PROMs used by participants is provided in Appendix S2.

**TABLE 7:** Patient Report Measures (PROMs) used routinely (*N* = 103).

	*N* (%)
0–10 Numeric Rating Scale^a^	97 (94%)
Functional Disability Inventory^a^	47 (46%)
PedsQL physical functioning	45 (44%)
PedsQL emotional functioning	32 (31%)
PedsQL school functioning	31 (30%)
PedsQL social functioning	27 (26%)
PROMIS Paediatric Depressive Symptoms 8a^a^	25 (24%)
Bath Adolescent Pain Questionnaire—Physical Functioning Scale^a^	25 (24%)
Adolescent Sleep Wake Scale—Short Form^a^	24 (23%)
PROMIS Paediatric Anxiety 8a^a^	23 (22%)
Child Activity Limitations Interview—Short form^a^	17 (17%)
Patient Global Impression of Change^a^	15 (15%)
Children's Depression Inventory—Short form^a^	15 (15%)

Abbreviations: PedsQL, Paediatric Quality of Life Inventory; PROMIS, Patient‐Reported Outcomes Measurement Information System paediatric measure.

^a^ Suggested PROMs by Palermo et al. (2024).

## 4 Discussion

This study identified the clinical outcome domains and measures used in clinical practices treating children and adolescents with chronic pain, highlighted the domains considered mandatory, and compared priorities between professions and countries. The findings provide important insights for developing a COS of outcome domains and measures to be used in clinical settings treating children and adolescents with chronic pain worldwide.

Not surprisingly, we found that pain intensity was the domain most commonly assessed by this sample of clinicians. Three additional domains that were also reported by a large majority (i.e., ≥ 75% of the study clinicians) were pain interference, physical functioning and pain severity. They were also considered mandatory domains by most of the participants, underscoring their central role in clinical practice. However, significant differences were observed between country income categories. For example, pain intensity was deemed mandatory by all clinicians from lower‐middle‐income countries and nearly all participants from upper‐middle‐income countries, exceeding the rates in high‐income countries. This finding highlights the universal recognition of pain intensity as a critical outcome, particularly in resource‐limited settings. In such contexts, limited time, staff and resources likely make pain intensity the most feasible and immediate indicator of treatment need and response. Moreover, significant differences were observed between country income categories; specifically, clinicians from high‐income countries consistently reported assessing more outcome domains compared to clinicians from upper‐middle‐ and lower‐middle‐income countries. For example, psychological functioning and sleep quality were measured more in high‐income countries. These observed differences may reflect the impact of national incomes on healthcare spending, with resultant financial and human resource pressures leading to the use of fewer domains and briefer measures. An additional practical issue concerns the cost and accessibility of assessment tools. Some commonly used instruments, such as the Children's Depression Inventory, require purchasing from a publisher, which may restrict their use in routine clinical settings and particularly in low‐ and middle‐income countries. Cost and licensing barriers represent a significant obstacle to the implementation of standardised assessment. The development and cross‐cultural validation of free, open‐access measures should therefore be prioritised to promote equitable clinical practice and research worldwide. A related ethical consideration concerns the administration of mental health measures in busy clinical settings. When such tools identify elevated levels of emotional distress, clinicians have a professional responsibility to respond appropriately, ideally with the support or referral of a mental health professional. Although our survey did not capture information about how clinicians handle these situations, future studies should investigate how psychological screening results are managed to ensure appropriate and ethical patient care.

Beyond addressing individual domains and measures, harmonising assessment practices across clinics offers substantial benefits. The use of shared, standardised tools would allow comparisons across sites, facilitate benchmarking and support the creation of collaborative (de‐identified) data registries. Such initiatives could enhance data quality, accelerate knowledge translation and strengthen evidence‐based paediatric pain care globally. This goal aligns with the ongoing objectives of the IN‐ChildPain Network to develop a consensus‐based paediatric Core Outcome Set of domains and respective measures for clinical use, ensuring that the most important outcomes with best suited measures are assessed, allowing benchmarking of results and improvement of treatment in the future.

Over half of participants reported that they used available PROMs for assessing certain domains, with higher adoption rates in high‐income countries. These findings align with previous research indicating greater implementation of PROMs in resource‐rich settings due to better access to training and infrastructure (Malapati et al. 2024; Masyuko et al. 2021). The 0–10 numerical rating scale (0–10 NRS) for the assessment of pain intensity was the only widely used PROM, a finding consistent with Connolly et al.'s (2019) review. This reliance on the 0–10 NRS may reflect its simplicity and language availability (Castarlenas et al. 2017), ensuring widespread applicability (Birnie et al. 2019). Other PROMs were reported to be used by fewer than half of the sample, and some were used by less than a quarter. The limited use of PROMs measuring other domains in lower income countries may reflect that the PROMs in the list provided to participants were either not available or validated in their native language, not available as free open‐access versions or that participants found them to be of little clinical utility.

The findings showed both consensus and divergence in the prioritisation of outcome domains as mandatory between individuals representing different professional groups. Domains such as pain intensity, pain interference and physical functioning were consistently valued, whereas domains such as treatment‐related adverse events, economic factors and cognitive functioning showed significant variability. Notably, there were differences in opinions about the importance of monitoring adverse events. These findings align with a recent review examining the assessment of adverse events in psychological interventions for children with mental health problems. Of the 117 studies analysed, only 36 studies (31%) monitored adverse events and just over half (53%) followed a protocol for this monitoring (Lodewyk et al. 2023). However, recording treatment adverse events ensures patient safety, informs clinical decisions and improves intervention effectiveness by identifying potential risks and optimising therapeutic approaches for children and adolescents with chronic pain. Accordingly, many COS for adults with acute and chronic pain as well as the COS for paediatric trials related to chronic pain include adverse events as mandatory (Duffy et al. 2020; Palermo et al. 2021; Pogatzki‐Zahn et al. 2021). The discrepancy between our survey's findings and Palermo et al.'s recommendations may reflect both the different methodologies employed and the distinct objectives and stakeholders involved.

The survey findings showed some concordance with previous similar studies, but also showed some significant differences. For example, some domains included by Palermo et al. (2021) among the core outcomes for paediatric chronic pain trials (i.e., pain intensity, physical functioning, emotional functioning, sleep and adverse events) were categorised as optional by a majority of the participants in this survey. Additionally, the participants in this survey identified some domains as mandatory that were not included as mandatory or optional for clinical trials (Palermo et al. 2021), indicating potential areas for further exploration. Unlike the Palermo et al. framework, which proposed a distinction between universally required (‘core’) and context‐dependent (‘optional’) domains, our survey did not reveal a consistent tiered structure among clinicians. Pain intensity, which is commonly considered a specific subdomain of pain severity (Jensen et al. 2024; Palermo et al. 2021), was the most used and valued domain by participants in this survey. Because perception of the severity of pain includes how intense the pain is and how frequently it occurs, more research is needed to identify what is most important for clinical practice. There were also significant discrepancies in relation to the PROMs used in clinical *practice* by participants reported in this survey, compared to those suggested by initiatives for clinical *trials* (Palermo et al. 2024). The findings from this survey could reflect that PROMs best suited for research purposes may not have adequate clinical utility. It may also signal differences related to healthcare providers' professional background or country income level. This highlights the potential need for harmonising PROMs within a COS to address the specific priorities and challenges of clinical settings.

These findings have several implications for clinical practice, education and research. First, the diversity in the prioritisation of pain‐related domains and the limited number of PROMs used highlights the need for standardising the domains to be assessed (COS of domains) and the appropriate measures (COS of PROMs) for clinical routine in paediatric chronic pain. The use of PROMs at the individual patient level, especially for monitoring symptoms and guiding care pathways, has been shown to improve health outcomes (Bonsel et al. 2024). Therefore, research is needed to translate and culturally adapt those PROMs best suited as psychometric measures or develop new ones when they are not available to assess those domains defined in a COS of domains for clinicians. Efforts to develop culturally relevant and resource‐appropriate outcome measures are essential to ensure equitable care.

The disparities in outcome domain assessment and PROMs usage across country income categories and professionals highlight the importance of addressing education and training gaps. Research is needed to study the effectiveness of clinician education programmes in improving understanding and implementation of mandatory domains. This, however, needs first a consensus on a COS of domains for paediatric chronic pain in the clinical setting.

The relatively low prioritisation of some domains (e.g., sleep quality) by the participants of our survey need further investigation by including the right balance of appropriate stakeholders, including patient representatives for their integration into a COS for clinical practice. Moreover, studies are needed to investigate how resource availability, cultural factors and healthcare infrastructure influence priorities for chronic pain assessment and treatment in children and adolescents. For example, an additional potential avenue for reducing pain management disparities globally might lie in finding or developing valid short‐form measures that fit better into pressured work flows, in order to define a culturally adapted COS of domains and later PROMs.

This study has several limitations that should be considered when interpreting the findings. First, the survey was intentionally designed to be brief in order to maximise completion rates, which resulted in limited collection of clinician background information. As a result, our ability to fully interpret and contextualise respondents' answers is restricted. Second, although professionals from all five continents participated, only a small proportion were from lower‐middle‐income countries, and none were from low‐income countries. This limits the generalisability of our findings to resource‐constrained settings. Third, the data reflect clinicians' perceptions only. Future studies addressing the development of a clinical COS should include representatives of all stakeholder groups, including patients, parents and health authorities. Fourth, although the survey identified mandatory domains of those participating in the survey, the data does not allow for detailed insights into the reasons that specific domains are prioritised in different countries and/or professional specialties, potentially oversimplifying complex cultural and systemic factors. Fifth, although multiple clinicians from the same programme could respond, all entries were manually checked for duplicates using programme names and demographics. Nevertheless, some degree of overrepresentation across clinics may still have occurred.

Despite the study's limitations, the findings provide valuable new insights into the clinical outcome domains that are considered mandatory by clinicians worldwide. Importantly, clinicians' prioritisation of domains and the use of PROMs found in this study could explain, at least in part, why PedIMMPACT suggestions have not been fully incorporated in clinical activities after all these years (Connolly et al. 2019). It remains to be determined whether the latest COS for clinical trials (Palermo et al. 2021) and suggested PROMs (Palermo et al. 2024) will have clinical utility. The findings of this survey highlight the need for a specific COS tailored to the clinical management of children and adolescents with chronic pain, one that considers the context in which it is to be used, particularly the healthcare system, and the cultural and linguistic particularities of the location.

## Funding

This study was facilitated by the International Network on Chronic Pain in Childhood (IN‐ChildPain), which was funded by the Network of European Funding for Neuroscience Research (ERA‐NET Neuron Transnational Networking Groups on Chronic Pain 2022) through the BMBF (grant reference 01EW2311).

## Conflicts of Interest

The authors declare no conflicts of interest.

## Supporting information


**Appendix S1:** ejp70216‐sup‐0001‐AppendixS1.docx.

**Appendix S2:** ejp70216‐sup‐0002‐AppendixS2.docx.

**Appendix S3:** ejp70216‐sup‐0003‐AppendixS3.doc.
