# Supplementary material for: Outcome Assessment in Children and Adolescents With Chronic Pain: An International Clinical Practice Survey
Source: Eur J Pain. 2026 Jan 17;30(1):e70216. doi: 10.1002/ejp.70216 (PMC12811898; doi:10.1002/ejp.70216)
Supplement: Supplementary file 1 — Appendix S1: ejp70216‐sup‐0001‐AppendixS1.docx. [file EJP-30-0-s001.docx]

**Supplementary data 1. Survey questions.**

**Introduction-Basic information about the chronic pain clinic/program:**

**Instructions**: Please respond to the following questions pertaining to the chronic pain clinic/program that you are currently associated with.

1. Identify the country where your chronic pain clinic/program is located:

1. Do your services include (check all that apply):
   1. An inpatient intensive chronic pain rehabilitation program
   2. A service associated with a hospital
   3. An outpatient Service in a (private) practice
   4. Others - please specify:
2. What is the name of your chronic pain clinic/program?
3. What is your profession?
   1. Anesthesiology
   2. General practitioner
   3. Psychology
   4. Physiotherapy
   5. Nursing
   6. Occupational therapy
   7. Psychiatry
   8. Other – please specify:

***Outcome Domain***: What should be measured, that is, “the outcomes”

***Measurement Instrument***: How to measure the outcome.

***Patient-reported outcome measure (PROM)***: A type of measurement instrument directly reported by the patient, often using self-report questionnaires

**Domains and Questionnaires:**

1. Which specific Outcome Domains does **your program/clinic/service** assess using self-report questionnaires in children and adolescents with chronic pain? Please, choose all that apply from the following list:
2. Pain intensity
3. Pain interference with daily living (i.e., how much pain interferes with engagement in social, physical, and recreational activities).
4. Pain severity (i.e., perception of the severity of pain including how intense is and how frequently it occurs).
5. Overall well-being (i.e., perception of overall -global- well-being; e.g., satisfaction with health and life).
6. Sleep (i.e., quantity and quality of sleep; e.g., problems with falling sleep).
7. Physical functioning (i.e., ability to perform physical activities).
8. Emotional functioning (i.e., psychological and emotional well-being; e.g., experiencing anxiety or depression).
9. Cognitive functioning (i.e., the mental processes involved in the acquisition of knowledge, manipulation of information, and reasoning; e.g., memory, attention span)
10. Fatigue (i.e., mental or physical tiredness)
11. Role functioning (i.e., the roles that a child/adolescent may enact; e.g., being a student, friend, employee, and family member).
12. Adverse events (i.e., an unwanted symptom or reaction from a treatment; e.g., stomach upset, vomiting, fatigue, etc.).
13. Economic factors (i.e., examination of both direct and indirect economic costs associated to chronic pain management).
14. Global judgment of treatment satisfaction (i.e., the level of satisfaction with treatment taking into account pain relief, side effects, physical recovery, emotional recovery, and economic considerations).
15. Others: please specify:
16. Identify which domains you believe should be “**mandatory**” (i.e., that all chronic pain programs/clinic/services should assess) or “**optional**” (i.e., that might just be relevant under specific circumstances; e.g., pain conditions or research purposes) for **clinical work.** Please, choose all that apply from the following list:
17. Pain intensity
18. Pain interference with daily living (i.e., how much pain interferes with engagement in social, physical, and recreational activities).
19. Pain severity (i.e., perception of the severity of pain including how intense is and how frequently it occurs).
20. Overall well being (i.e., perception of overall -global- well-being (e.g., satisfaction with health and life).
21. Sleep (i.e., quantity and quality of sleep; e.g., problems with falling sleep).
22. Physical functioning (i.e., ability to perform physical activities).
23. Emotional functioning (i.e., psychological and emotional well-being; e.g., experiencing anxiety or depression).
24. Cognitive functioning (i.e., the mental processes involved in the acquisition of knowledge, manipulation of information, and reasoning; e.g., memory, attention span)
25. Fatigue (i.e., mental or physical tiredness)
26. Role functioning (i.e., the roles that a child/adolescent may enact; e.g., being a student, friend, employee, and family member).
27. Adverse events (i.e., an unwanted symptom or reaction from a treatment; e.g., stomach upset, vomiting, fatigue, etc.).
28. Economic factors (i.e., examination of both direct and indirect economic costs).
29. Overall judgment of treatment satisfaction (i.e., the level of satisfaction with treatment taking into account pain relief, side effects, physical recovery, emotional recovery, and economic considerations).
30. Others: please specify:
31. Are there standardized questionnaires (or Patient Reported Outcome Measures, PROMs) that your program routinely uses for evaluating children and adolescents with chronic pain? If yes, please, choose all that apply from the following:
32. 0-10 Pain intensity Numerical Rating Scale
33. Functional Disability Inventory
34. Pediatric Quality of Life _PedsQL-Physical function domain
35. Child Activity Limitations Interview Short Version (CALI-9)
36. Patient Global Impression of Change
37. PROMIS Pediatric Depressive Symptoms 8a
38. PROMIS Pediatric Anxiety 8a
39. Children’s Depression Inventory Short version
40. Pediatric Quality of Life _PedsQL-Emotional functioning domain
41. Pediatric Quality of Life _PedsQL-Social functioning domain
42. Pediatric Quality of Life _PedsQL-School functioning domain
43. Bath Adolescent Pain Questionnaire Physical Functioning Scale
44. Adolescent Sleep Wake Scale Short Form
45. What other standardized questionnaires do you use that are not included in the previous list? If the questionnaire is in your local language, can you let us know the name?

A **pain registry** is a standardized approach to collect data that is used for a variety of purposes, including, clinical- scientific-, and policy-related.

1. Does your clinic/program participate in a chronic pain registry? If yes, please specify the name of the registry
2. Would you be willing to be available for further inquiries or collaboration? If yes, please provide an email where we can contact you and inform you of future opportunities.
