# Supplementary material for: Outcome Assessment in Children and Adolescents With Chronic Pain: An International Clinical Practice Survey
Source: Eur J Pain. 2026 Jan 17;30(1):e70216. doi: 10.1002/ejp.70216 (PMC12811898; doi:10.1002/ejp.70216)
Supplement: Supplementary file 2 — Appendix S2: ejp70216‐sup‐0002‐AppendixS2.docx. [file EJP-30-0-s003.docx]

**Supplementary data 2. List of all PROMs used as reported by participants.**

1. Bath Adolescent Pain Questionnaire (entire questionnaire for children and parents) (BAPQ)
2. Beck Depression Inventory (BDI)
3. Behaviour Assessment System (BASC-III)
4. Brief Pain Inventory (BPI)
5. Center of Epidemiologic Studies Depression scale (CES_DC)
6. Central Sensitization Inventory (CSI)
7. Child Acceptance and Mindfulness Measure (CAMM)
8. Child Behaviour Checklist (CBCL)
9. Child Health Assessment Questionnaire (CHAQ)
10. Childhood Health Assessment Questionnaire (CHAQ)
11. Chronic Pain Acceptance Questionnaire – Adolescents (CPAQ-A)
12. Chronic Pain Grading Scale (CPGS)
13. Detail and Flexibility Questionnaire – Short Form (DFLEX-SF)
14. Disease Activity Score – 28 (DAS-28)
15. Douleur Neuropathique – 4 (DN4)
16. Échelle Douleur Inconfort Nouveau-Né (EDIN-6)
17. Face, Legs, Activity, Cry, Consolability – Revised (FLACC-R)
18. Faces Pain Scale – Revised (FPS-R)
19. Fear of Pain Questionnaire for Children (FOPQ-C)
20. General Anxiety Disorder – 7 (GAD-7)
21. German version of the Pain Questionnaire for children and adolescents (DSF-KJ)
22. Juvenile Arthritis Disease Activity Score (JADAS)
23. Lower extremity functional Scale (LEFS)
24. Michigan Body Map
25. Moods and Feelings Questionnaire (MFQ)
26. Non-communicating Children's Pain Checklist – Revised (NCCPC-R)
27. Opioid Risk tool (ORT)
28. Pain Catastrophizing Scale – Children (PCS—C)
29. Pain coping skills questionnaire for parents and children
30. PainDetect
31. Parental Pain Catastrophizing Scale (PCS-P)
32. Patient Health Questionnaire – 9 (PHQ-9)
33. Pediatric Pain Disability Index (P-PDI)
34. Photograph Series of Daily Activities for youth (PHODAS-Youth)
35. PROMIS Fatigue
36. PROMIS mobility
37. PROMIS pain interference
38. PROMIS Psychological Stress experiences
39. PROMIS sleep disturbance
40. Revised Children’s Anxiety and Depression Scale (RCADS)
41. Self-Compassion Scale – Short Form (SCS-SF)
42. Silhouettes Fatigue Scale (SFS)
43. Survey of Pain Attitudes – Pediatric form (PedSOPA)
44. Tampa Scale for Kinesiophobia (TSK-11)
45. Visual Analogue Scale (VAS)
46. Youth Self-report (YSR)
